# Long‐Term Clinical and Psychological Efficacy and Safety of Ocrelizumab in People With Multiple Sclerosis: A Real‐World Longitudinal Study

**DOI:** 10.1002/hsr2.72142

**Published:** 2026-03-22

**Authors:** Majid Jafari, Mohammad Yazdan Panah, Saeed Vaheb, Iman Adibi, Fereshteh Ashtari, Vahid Shaygannejad, Aram Zabeti, Omid Mirmosayyeb

**Affiliations:** ^1^ Clinical Research Development Unit, Kashani Hospital Shahrekord University of Medical Sciences Shahrekord Iran; ^2^ Isfahan Neurosciences Research Center Isfahan University of Medical Sciences Isfahan Iran; ^3^ Department of Neurology Isfahan University of Medical Sciences Isfahan Iran; ^4^ Department of Neurology University of Cincinnati Cincinnati OH USA

**Keywords:** efficacy, longitudinal, Multiple sclerosis, ocrelizumab, real‐world, safety

## Abstract

**Background:**

Multiple sclerosis (MS) is the most prevalent immune‐mediated neurodegenerative disease affecting the central nervous system. Ocrelizumab, a humanized anti‐CD20 monoclonal antibody, has demonstrated significant efficacy in reducing disease activity and improving clinical outcomes of people with MS (PwMS) in clinical trials. However, real‐world data are crucial for assessing the efficacy and safety of ocrelizumab. Thus, the current study aimed to evaluate the long‐term clinical and psychological efficacy and safety profile of ocrelizumab in PwMS compared with other disease‐modifying therapies (DMTs).

**Methods:**

This longitudinal study was carried out between January 2022 and February 2024 in Isfahan, Iran. 51 PwMS were included, of whom 21 patients were DMT‐naïve, while 30 patients were on other DMTs, including interferon‐β, fingolimod, dimethyl fumarate, natalizumab, or teriflunomide. Demographic characteristics, clinical outcomes, including Expanded Disability Status Scale (EDSS), annualized relapse rate (ARR), timed 25‐foot walk (T25FW) test, and nine‐hole peg test (9‐HPT), as well as psychological measures including anxiety, depression, and fatigue, were evaluated at baseline (T0), and 12 months (T1) and 24 months (T2) of follow‐up. Adverse events (AEs) were obtained using chart review and patient self‐reports in clinical visits.

**Results:**

ARR was significantly decreased at T1 (Z = −5.4, *p* < 0.001) and T2 (Z = −4.03, *p* < 0.001) in PwMS who received ocrelizumab. Similarly, a significant decrease was found in 9‐HPT (Z = −5.16, *p* < 0.001) and T25FW (Cohen's d = −0.72, *p* < 0.001) at T1. Depression and fatigue significantly improved at T1 (Z = −3.89, *p* < 0.001, Cohen's d = −1.16, *p* < 0.001, respectively) and further at T2 (Z = −2.18, *p* = 0.029, Cohen's d = −1.59, *p* < 0.001, respectively), and anxiety at T1 (Z = −3, *p* = 0.003). PwMS who initiated ocrelizumab as a first‐line therapy showed a significantly greater reduction in ARR, 9‐HPT, and T25FW than those previously treated with other DMTs (all *p* < 0.05), while no significant differences were observed in terms of changing EDSS, anxiety, depression, or fatigue levels. Respiratory infections (13.7%), infusion‐related reactions (5.9%), and headache (5.9%) were the most frequent AEs, and no serious infection was observed.

**Conclusion:**

Ocrelizumab may reduce disease activity and improve functional and psychological outcomes in PwMS, while maintaining a favorable safety profile. Moreover, relapse rates and functional impairment were improved more in groups that initiated ocrelizumab as a first‐line therapy than in other disease‐modifying therapies. These findings suggest that ocrelizumab is a promising first‐line treatment in MS. However, further studies are needed to confirm these preliminary results.

## 1 Introduction

Multiple sclerosis (MS) is a chronic immune‐mediated disease of the central nervous system (CNS) that can cause gradual worsening of neurological function [1]. Approximately 85% to 90% of people with MS (PwMS) present with the relapsing–remitting (RRMS) phenotype, which is characterized by recurrent episodes of neurological dysfunction followed by partial or complete recovery, with periods of remission between attacks [2]. Within about a decade, nearly 25% of RRMS patients progress to progressive MS (PMS), defined by continuous neurological decline, with or without relapses [2]. Although current therapies effectively reduce or prevent relapses in RRMS patients, they show limited efficacy in controlling relapse‐independent progression and offer minimal benefit for PMS patients [3].

Although multiple sclerosis (MS) was previously characterized as a predominantly T cell‐mediated demyelinating disorder [4], accumulating evidence has revealed the critical contribution of B cells, identifying the CD20 surface molecule as a significant therapeutic target [5]. CD20 is a non‐glycosylated transmembrane phosphoprotein expressed on B lymphocytes during most stages of maturation, except pro‐B cells and terminally differentiated plasma blasts and plasma cells [6]. Additionally, CD20 expression has been identified on a small subset of circulating T cells [7]. At present, five anti‐CD20 therapies, including rituximab, ofatumumab, ublituximab, BCD‐132, and ocrelizumab, have been approved or are under clinical investigation for the treatment of MS [4].

Ocrelizumab is a recombinant humanized monoclonal antibody that selectively targets and depletes CD20‐positive B cells [8]. It has been shown to significantly improve clinical outcomes, including reductions in annualized relapse rates (ARR) and a reduction in disability progression [9, 10, 11, 12]. In addition to neurological outcomes, psychological symptoms represent a fundamental component of the overall disease burden in MS. Fatigue is among the most common and disabling symptoms of MS and affects more than half of PwMS [13]. Depression and anxiety are also highly prevalent among PwMS and lead to difficulties in daily functioning, poor treatment adherence, and decreased quality of life [14, 15]. Additionally, cognitive impairment affects nearly 30% of PwMS and adversely impacts their quality of life and occupational functioning [16, 17]. Ocrelizumab can improve health‐related quality of life [18, 19], reduce fatigue [20], stabilize depression [15], and enhance cognitive performance [21]. Although randomized controlled trials (RCTs) provide strong evidence of its therapeutic efficacy, their participants are not often an equivalent sample in real‐world settings [8]. Therefore, real‐world studies are helpful to reassess the effectiveness of ocrelizumab comprehensively [8].

Although anti‐CD20 therapies such as ocrelizumab can reduce disease activity in MS [22, 23], their mechanism can increase the risk of infections [24, 25]. The most common adverse events (AEs) after ocrelizumab are mild respiratory and urinary tract infections. Serious infections are rare, though careful monitoring and timely management are recommended [24, 25]. Real‐world evidence linked longer ocrelizumab exposure to a higher rate of serious infections than with injectables [26]. Rare malignancies have been reported following ocrelizumab treatment. These observations underscore the importance of careful monitoring in PwMS receiving ocrelizumab [22, 27]. Therefore, both the therapeutic benefits and potential safety risks of ocrelizumab should be weighed together. This highlights the need for real‐world studies to more clearly define its suitability as a first‐line treatment [22, 25].

Evidence from both real‐world studies and RCTs is essential for making treatment plans [28]. The long‐term effects of ocrelizumab in PwMS are being evaluated through ongoing research [29, 30]. Real‐world studies continue to fill these knowledge gaps and provide valuable evidence for the therapeutic effectiveness of ocrelizumab [20, 31]. However, the long‐term and real‐world effects of ocrelizumab on the clinical and psychological characteristics of PwMS have not been adequately explored. Thus, the present study aimed to investigate the longitudinal effects of ocrelizumab on functional performance and psychological features of PwMS, as well as comparing these outcomes between ocrelizumab naïve patients and switchers to ocrelizumab from other DMTs.

## 2 Methods

### 2.1 Study Design and Participants

This cohort study was conducted on PwMS who initiated ocrelizumab between January 2022 and February 2024, and used clinical data from the Isfahan Hakim MS Database (IHMSD) [32] in Isfahan, Iran. PwMS were consecutively enrolled after being prescribed ocrelizumab in line with the FDA‐approved indications for MS treatment. Participants were eligible if they were over 18 years old, followed the treatment protocol, had a confirmed diagnosis of MS [33], completed at least four cycles of ocrelizumab, and had undergone a minimum of three Expanded Disability Status Scale (EDSS) [34] assessments. Participants were classified into one of two disease phenotypes, RRMS or PMS [35], at the time of initiation of treatment with ocrelizumab. Follow‐up was maintained until death, emigration, treatment discontinuation, or the date of data extraction. Exclusion criteria encompassed individuals below 18 or above 55 years of age, pregnancy, brain mass lesions, malignancies, autoimmune diseases other than MS, active infections, endocrinological disorders, clinically diagnosed anxiety or depression, those receiving antidepressant or anxiolytic medications, corticosteroid treatment within the preceding 3 months, and menopausal status. All PwMS who fulfilled the inclusion criteria and either initiated or switched to ocrelizumab during the recruitment period were enrolled.

**Figure 1:**
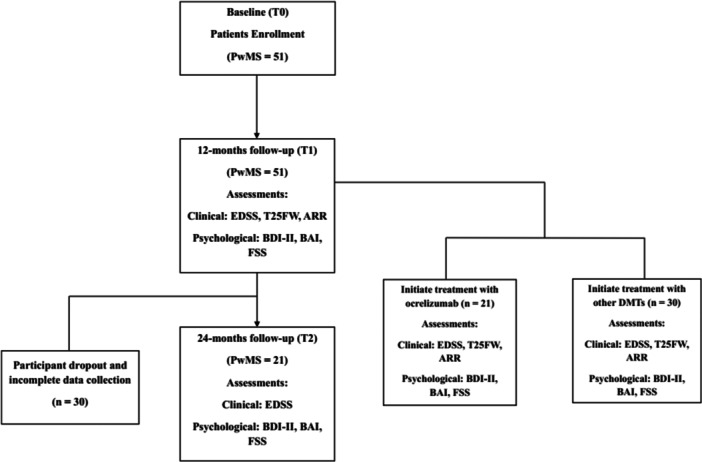
Flow diagram of the study process.

A total of 51 PwMS were enrolled in the study. All participants were evaluated at baseline (T0) and at 12 months (T1), whereas 20 participants completed the 24‐month follow‐up (T2). The reduction in sample size at T2 was due to loss to follow‐up and incomplete data (Figure 1). Baseline demographic, clinical, and psychological data were collected from PwMS, including age, age at disease onset, gender, marital status, education level, MS phenotype, disease duration, number of ocrelizumab cycles, prior DMTs, anxiety, depression, and fatigue. The sociodemographic variables were included to provide a comprehensive characterization of the study population. An expert neurologist evaluated the EDSS, timed 25‐foot walk (T25FW) test, and nine‐hole peg test (9‐HPT). Among the RRMS phenotypes, data were gathered on the total number of relapses since diagnosis, the occurrence and frequency of relapses within the previous year, and the duration (in months) since the most recent relapse.

AEs were recorded through structured chart review and patient self‐report during routine clinical visits, with clinical evaluation performed by the treating neurologist. Infusion‐related reaction (IRR) is defined as any AEs that occurred during infusion or within 24 h after the course finished [36]. Symptoms such as headache, flushing, erythema, and pruritus were counted as IRRs when they fell within this window. Events arising after 24 h were recorded separately as AEs. To assess perceived severity, patients rated each AE on a 5‐point Likert scale (1 = not at all; 2 = a little bit; 3 = somewhat; 4 = quite a bit; 5 = very much) [37].

Ultimately, PwMS were classified into two groups based on their DMT history: (a) those who started treatment with ocrelizumab and (b) those who initially received other DMTs but switched to ocrelizumab at the start of the study. The group of previously treated PwMS included those who had received interferon‐β, fingolimod, dimethyl fumarate, natalizumab, or teriflunomide before switching to ocrelizumab. The follow‐up period was defined as the interval between the initial infusion and the final evaluation date.

### 2.2 Sample Size Determination

The sample size was estimated based on standard formulas for detecting mean differences between two independent or paired groups. For continuous outcomes, the minimum required sample size per group is given by:

$$n=\frac{2\times {\;(z\left(1-\frac{\alpha }{2}\right)+z\left(1-\beta \right))}^{2}}{d^{2}}$$

Where d is the standardized effect size (Cohen's d), α is the two‐sided significance level, and 1−β is the desired power. Assuming a moderate effect size (d = 0.62), significance level α = 0.05, and statistical power of 80%, the minimum required sample size is 21 individuals.

### 2.3 Protocol of Ocrelizumab Infusion

Ocrelizumab was administered according to its standard regimen: an initial dose of 600 mg, split into two 300 mg infusions 2 weeks apart, followed by single 600 mg infusions every 6 months. Before initiation of the infusion, each patient was examined for symptoms of infection, including SARS‐CoV‐2 and influenza. Premedication with corticosteroids, antihistamines, and antipyretics is considered to help reduce the risk of IRRs. Infusions were administered over at least 2.5 h for the 300 mg dose and 3.5 h for the 600 mg dose. Then, PwMS were observed for 1 h to monitor vital signs. PwMS who exhibited no adverse reactions during this period were discharged.

### 2.4 Outcomes

EDSS, ARR, depression, anxiety, and fatigue were assessed at T0, T1, and T2. Additionally, upper limb function and ambulation speed using the 9‐HPT [38] and the T25FW test [39], were measured at T0 and T1.

A relapse was defined as a recurrence or new onset of neurological symptoms lasting more than 24 h and should occur at least 30 days after a previous episode and not be associated with fever or infection [40, 41]. Diagnosis of relapse was performed by an experienced neurologist during neurological examination and documented at the time of evaluation. The ARR was calculated using the person‐years method by dividing the total number of relapses by the cumulative person‐years of follow‐up for the study population [42, 43]. To ensure inter‐rater reliability, the same expert neurologist conducted the EDSS, T25FW, and 9‐HPT assessments at each visit. When a relapse occurred at or near a scheduled visit, we repeated the assessment 30 days after symptoms had resolved, once the patient was relapse‐free and clinically stable. This practice helped limit bias from short‐term impairments related to the relapse.

### 2.5 Beck Depression Inventory‐II (BdI‐II)

The Beck Depression Inventory‐II (BDI‐II) is a self‐reported questionnaire that assesses depressive symptoms across cognitive, physiological, emotional, and motivational domains [44]. It consists of 21 items that evaluate symptoms such as agitation, feelings of worthlessness, concentration difficulties, and fatigue. Each item consists of four statements that represent increasing levels of symptom severity, rated on a scale from 0 to 3 [44]. The BDI‐II has been validated in the Persian language [45].

### 2.6 Beck Anxiety Inventory (BAI)

The Beck Anxiety Inventory (BAI) is a 21‐item and self‐report instrument to evaluate the presence and severity of anxiety symptoms [46]. The total score ranges from 0 to 63, with higher scores indicating greater levels of anxiety. Severity levels are classified as mild (8 to 15 scores), moderate (16 to 25 scores), and severe (26 to 63 scores) [46]. The Persian version of this questionnaire has the validity and reliability coefficients of 0.72 and 0.83, respectively [47].

### 2.7 Fatigue Severity Scale (FSS)

The Fatigue Severity Scale (FSS) is a nine‐item and self‐report questionnaire that evaluates the intensity of fatigue and its effects on daily functioning and lifestyle over the past week. Each item is scored on a 7‐point Likert scale, with scores of 4 or higher reflecting a significant impact of fatigue. Higher total scores indicate greater fatigue severity and functional impairment [48, 49]. The Persian version of the FSS has shown reliability and validity among PwMS [50].

### 2.8 Statistical Analysis

Statistical analyses were conducted using IBM SPSS Statistics version 26 for Windows (IBM Corp., Armonk, NY, USA). Sociodemographic data were summarized using descriptive statistics. The normality of data distribution was examined with Q‐Q and P‐P plots, as well as the Shapiro–Wilk test. Results were reported as frequencies (*n*), percentages (%), means (standard deviation (SD)), or medians with range. Group comparisons were performed using the independent samples *t*‐test for normally distributed variables and the Mann–Whitney *U* or Wilcoxon tests for non‐normally distributed data. Subgroup and stratified analyses were further applied to explore the effect of ocrelizumab in different clinical groups. Between‐group comparisons by initial therapy (ocrelizumab‐initiating vs. switching from other DMTs) were conducted as unadjusted exploratory analyses due to the limited sample size. All primary outcomes and time‐point comparisons were predefined. Therefore, no formal correction for multiple comparisons was applied. The limited number of statistical tests and the longitudinal within‐subject design reduced the likelihood of Type I error. Given the limited sample size, multivariable adjustment for all potential confounders was not feasible. Therefore, subgroup analyses were conducted according to treatment initiation type (ocrelizumab vs. other DMTs). Analyses were performed using a complete‐case approach, with no imputation for missing data. Results were evaluated with a 95% confidence interval, and a significance level of *p* < 0.05 was applied.

## 3 Results

### 3.1 Study Population Characteristics

Fifty‐one PwMS, each having received at least two ocrelizumab infusions within a year, were included in the study. The study population had a mean age of 37.3 years (SD 10.1), with 86.3% being female. The average disease duration was 7.8 years (SD 4.4), 41.2% were DMT‐naïve, and the rest of the PwMS were receiving interferon‐β before initiating ocrelizumab. The median EDSS score was 3 (range: 1.5 to 4.5). The details of baseline demographic, clinical, and psychological characteristics of PwMS are summarized in Table 1.

**Table 1:** Sociodemographic and clinical characteristics of people with multiple sclerosis.

Characteristics	*N* = 51
Female (%)	44 (86.3%)
Age (year); Mean (SD)	37.3 (10.1)
Age at Disease Onset (year); Mean (SD)	29.5 (9.5)
Marital Status; *n* (%)	
Single	10 (19.6%)
Married	41 (80.4%)
Year of Education; Mean (SD)	14.2 (2.6)
MS Phenotype; *n* (%)	
RRMS	32 (62.7%)
PMS	19 (37.3%)
Disease Duration (year); Mean (SD)	7.8 (4.4)
EDSS; Median (range)	3 (1.5 to 4.5)
Follow‐up Duration (months); Median (range)	25 (24 to 28)
DMT; *n* (%)	
Dimethyl fumarate	3 (10%)
Fingolimod	4 (13.3%)
Teriflunomide	1 (3.3%)
Interferon	21 (70%)
Natalizumab	1 (3.3%)
DMT Naïve at the Baseline	21 (41.2%)

*Note:* All quantitative data are presented as mean (SD) for normally distributed variables or median (range) for non‐normally distributed variables.

Abbreviations: DMT, disease‐modifying therapy; EDSS, Expanded Disability Status Scale; MS, multiple sclerosis; SD, standard deviation.

To evaluate potential attrition bias, baseline characteristics were compared between PwMS who completed the 24‐month follow‐up (*n* = 21) and those lost to follow‐up (*n* = 30). No significant differences were observed in age, sex distribution, MS subtype, disease duration, baseline EDSS, ARR, T25FW, 9‐HPT, or fatigue scores (all *p* > 0.05). Baseline anxiety showed a borderline difference (*p* = 0.052). However, baseline depression scores were significantly higher in PwMS lost to follow‐up (*p* = 0.011). More details are summarized in Supporting Material, Table S1.

### 3.2 Efficacy of Ocrelizumab

#### 3.2.1 Clinical Outcomes

**Figure 2:**
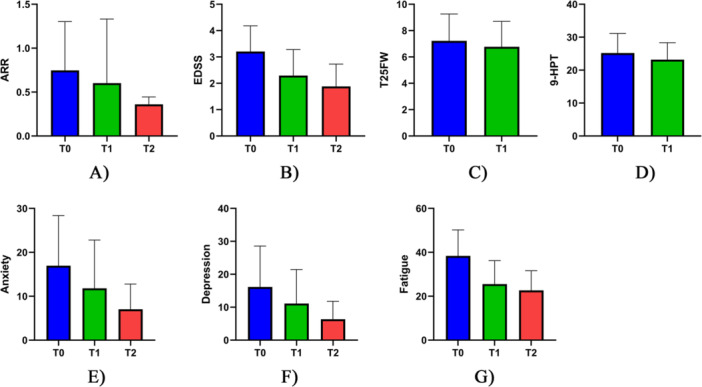
Comparing clinical and psychological characteristics of people with MS who were treated with ocrelizumab at baseline (T0), 12 months (T1), and 24 months (T2). (A) ARR, (B) EDSS, (C) T25FW, (D) 9‐HPT, (E) Anxiety, (F) Depression, and (G) Fatigue.

The median ARR demonstrated a significant reduction from 0.5 (range: 0.3 to 2) at T0 to 0.45 (range: 0.3 to 1) at T1 (Z = −5.4, *p*‐value < 0.001) and 0.3 (range: 0.27 to 0.67) at T2 (Z = −4.03, *p*‐value < 0.001). The median EDSS similarly decreased from 3 (range: 1.5 to 4.5) at T0 to 2 (range: 1 to 4) at T1 (Z = −5.64, *p*‐value < 0.001) and 1.5 (range: 1 to 3.5) at T2 (Z = −3.85, *p*‐value < 0.001). Additionally, following treatment with ocrelizumab, PwMS showed significant improvements in functional performance, with the median 9‐HPT reducing from 23.7 (range: 19.2 to 53.6) to 22.1 (range: 19.1 to 50.3) (Z = −5.16, *p*‐value < 0.001) and the mean T25FW test decreasing from 7.2 s (SD: 2) to 6.8 s (SD: 1.9) (Cohen's d = −0.72, *p*‐value < 0.001) at T1. More details of the clinical effectiveness of ocrelizumab in PwMS are outlined in Table 2 and Figure 2.

**Table 2:** Comparing clinical and psychological characteristics of people with MS at baseline, 12 months, and 24 months following treatment with ocrelizumab.

Characteristic	Baseline	12 Months	24 Months	Baseline versus 12 Months		Baseline versus 24 Months		12 Months versus 24 Months	
				Effect Size	*P*‐value	Effect Size	*P*‐value	Effect Size	*P*‐value
ARR; Median (range)	0.5 (0.3 to 2)	0.45 (0.3 to 1)	0.3 (0.27 to 0.67)	Z = −5.40	**< 0.001**	Z = −4.03	**< 0.001**	Z = −4.03	**< 0.001**
EDSS; Median (range)	3 (1.5 to 4.5)	2 (1 to 4)	1.5 (1 to 3.5)	Z = −5.64	**< 0.001**	Z = −3.85	**< 0.001**	Z = −2.41	**0.016**
9‐HPT; Median (range)	23.7 (19.2 to 53.6)	22.1 (19.1 to 50.3)	—	Z = −5.16	**< 0.001**	—	—	—	—
T25FW; Mean (SD)	7.2 (2)	6.8 (1.9)	—	Cohen's d = −0.72	**< 0.001**	—	—	—	—
Anxiety; Median (range)	15 (0 to 56)	5 (0 to 20)	5 (0 to 18)	Z = −3	**0.003**	Z = −3.5	**< 0.001**	−0.34	0.73
Depression; Median (range)	9 (1 to 47)	7 (0 to 28)	5 (0 to 17)	Z = −3.89	**< 0.001**	Z = −3	**0.002**	Z = −2.18	**0.029**
Fatigue; Mean (SD)	36 (11.3)	25.6 (10.1)	22.7 (9)	Cohen's d = −1.16	**< 0.001**	Cohen's d = −1.59	**< 0.001**	Cohen's d = 0.65	**< 0.001**

*Note:* Significant *p*‐values are shown in bold. Data are presented as mean (SD) for continuous variables and median (range) for non‐normally distributed variables.

Abbreviations: ARR, annualized relapse rate; EDSS, Expanded Disability Status Scale; 9‐HPT, nine‐hole peg test; MS, multiple sclerosis; SD, standard deviation; T25FW, time 25‐foot walk test.

#### 3.2.2 Psychological Outcomes

The findings revealed significant enhancements in the psychological well‐being of PwMS over one‐ and 2‐year periods after treatment with ocrelizumab. The median depression significantly declined from 9 (range: 1 to 47) at T0 to 7 (range: 0 to 28) at T1 (Z = −3.89, *p*‐value < 0.001) and 5 (range: 0 to 17) at T2 (Z = −2.18, *p*‐value = 0.029). Similarly, the median anxiety score decreased from 15 (range: 0 to 56) at T0 to 5 (range: 0 to 20) at T1 (Z = −3, *p*‐value = 0.003). Moreover, the mean level of fatigue exhibited a significant reduction from 36 (SD = 11.3) at T0 to 25.6 (SD = 10.1) at T1 (Cohen's d = −1.16, *p*‐value < 0.001) and 22.7 (SD = 9) at T2 (Cohen's d = −1.59, *p*‐value < 0.001). Further details of the psychological efficacy of ocrelizumab are summarized in Table 2 and Figure 2.

#### 3.2.3 Comparing Clinical and Psychological Outcomes of Initiation Treatment With and Without Ocrelizumab

To compare the clinical and psychological effects of initiating treatment with ocrelizumab or other DMTs, PwMS were stratified into two groups based on their initial treatment, and the clinical and psychological changes from T0 to T1 were analyzed and compared between these two groups. Baseline characteristics of patients initiating ocrelizumab and those switching from other DMTs are presented in Supplementary Table S1. The two groups did not differ significantly in most demographic or psychological variables; however, patients switching from other DMTs showed longer disease duration (*p*‐value < 0.001), lower baseline ARR (*p*‐value < 0.001), and slower T25FW performance (*p*‐value = 0.023) (Supporting Material, Table S2).

**Figure 3:**
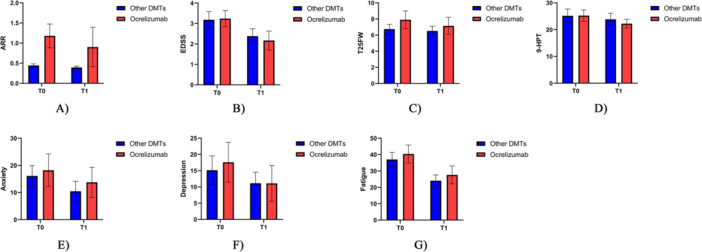
Comparing clinical and psychological characteristics of people with MS who were treated with ocrelizumab and those who were DMT Naïve (blue column) or treated with other disease‐modifying therapies (red column) before beginning the study at 12 months. T0 (Baseline), T1 (12 Months), (A) ARR, (B) EDSS, (C) T25FW, (D) 9‐HPT, (E) Anxiety, (F) Depression, and (G) Fatigue.

Regarding the clinical consequences, initiating ocrelizumab treatment led to significantly greater reductions in ARR (median (range) = −0.28 (−0.77 to −0.22) versus −0.05 (−0.06 to −0.04), Z = −3.71, *p*‐value < 0.001), 9‐HPT (median (range) = −3 (−4.3 to −1.7) versus −1.32 (−1.96 to −0.68), Z = −2.13, *p*‐value = 0.033), and T25FW test (median (range) = −0.76 (−1.11 to −0.4) versus −0.23 (−0.37 to −0.1), Z = −2.66, *p*‐value = 0.008) compared to other DMTs, while EDSS (median (range) = −1.07 (−1.39 to −0.75) versus −0.8 (−1 to −0.6), Z = −1.54, *p*‐value = 0.124) showed no significant difference. Moreover, regarding psychological consequences, there were no significant differences in anxiety (median (range) = −4.43 (−8.43 to −0.42) versus −5.63 (−10.13 to −1.13), Z = −0.04, *p*‐value = 0.969), depression (median (range) = −6.47 (−10.32 to −2.63) versus −4 (−7.77 to −0.23), Z = −1.21, *p*‐value = 0.226), and fatigue (median (range) = −12.76 (−18.23 to −7.3) versus −12.93 (−16.86 to −9), Z = −0.45, *p*‐value = 0.652) outcomes between initiate treatment with ocrelizumab and other DMTs (Table 3 and Figure 3).

**Table 3:** Between subject differences in clinical and psychological characteristics in people with MS who were treated with ocrelizumab and those who were DMT naïve or treated with other disease‐modifying therapies before beginning the study at 12 months.

	Mean Difference (95% Confidence Interval)			
	Initiate with OCR (*n* = 21)	Initiate with other DMTs (*n* = 30)	Effect Size	*p*‐value
ARR	−0.28 (−0.77 to −0.22)	−0.05 (−0.06 to −0.04)	MWU = 121.5 Z = −3.71	**< 0.001**
EDSS	−1.07 (−1.39 to −0.75)	−0.8 (−1 to −0.6)	MWU = 237 Z = −1.54	0.124
9‐HPT	−3 (−4.3 to −1.7)	−1.32 (−1.96 to −0.68)	MWU = 203.5 Z = −2.13	**0.033**
T25FW	−0.76 (−1.11 to −0.4)	−0.23 (−0.37 to −0.1)	MWU = 176 Z = −2.66	**0.008**
Anxiety	−4.43 (−8.43 to −0.42)	−5.63 (−10.13 to −1.13)	MWU = 313 Z = −0.04	0.969
Depression	−6.47 (−10.32 to −2.63)	−4 (−7.77 to −0.23)	MWU = 252 Z = −1.21	0.226
Fatigue	−12.76 (−18.23 to −7.3)	−12.93 (−16.86 to −9)	MWU = 291.5 Z = −0.45	0.652

*Note:* Significant *p*‐values are shown in bold.

Abbreviations: ARR, annualized relapse rate; DMTs, disease‐modifying therapies; EDSS, Expanded Disability Status Scale; 9‐HPT, nine‐hole peg test; MS, multiple sclerosis; OCR, ocrelizumab; SD, standard deviation; T25FW, time 25‐foot walk test.

#### 3.2.4 Safety of Ocrelizumab

**Figure 4:**
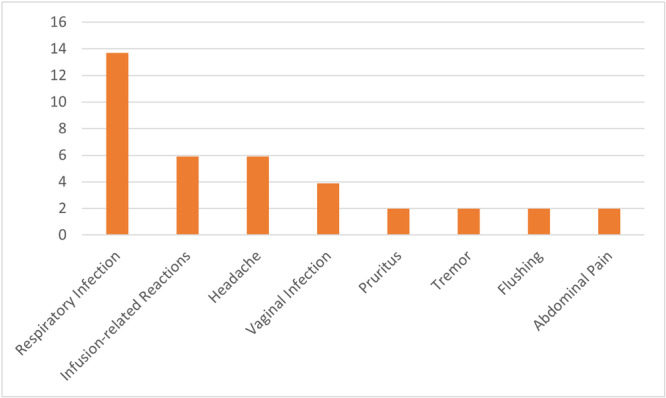
Frequency of adverse events following treatment with ocrelizumab in people with MS.

Respiratory infection was the most frequent adverse event during the 2‐year follow‐up (13.7%), followed by IRR (5.9), headache (5.9%), vaginal infection (3.9%), and while pruritus (1.9%), tremor (1.9%), flushing (1.9%), and abdominal pain (1.9%) were the least frequent. No treatment discontinuations occurred due to AEs (Table 4 and Figure 4).

**Table 4:** Frequency of adverse events following treatment with ocrelizumab in people with MS.

Side Effect	Frequency (%)	Mean Severity (Likert)
Respiratory Infection	7 (13.7%)	2.4
Infusion‐related Reactions	3 (5.9%)	2.7
Headache	3 (5.9%)	2.3
Vaginal Infection	2 (3.9%)	4.5
Pruritus	1 (2%)	2
Tremor	1 (2%)	2
Flushing	1 (2%)	3
Abdominal Pain	1 (2%)	3

## 4 Discussion

Due to the age and comorbidity restrictions in phase III trials, this study represents an MS cohort evaluating the extended real‐world follow‐up clinical outcomes, psychological benefits, and safety of ocrelizumab. Furthermore, this study explored potential differences in clinical and psychological outcomes between initiating treatment with ocrelizumab or other DMTs. The present study indicated extended real‐world follow‐up clinical and psychological benefits in ocrelizumab‐treated PwMS. This is consistent with ocrelizumab's primary pharmacological mechanism of action, which acts on the reduction of inflammatory activity within the central nervous system. Moreover, the absence of serious infections in this study supports the safety profile of ocrelizumab among PwMS.

Ocrelizumab mediates its immunomodulatory effects through the depletion of CD20 + B cells, reducing their number and function [51, 52]. In relapsing MS, the trafficking of activated oligoclonal B‐cell populations between the central nervous system and peripheral circulation has been identified [53, 54], with the disruption of this network potentially explaining ocrelizumab's mechanism of action [55]. Ocrelizumab is the most effective monoclonal antibody for progressive MS but is associated with an increased risk of infections [56]. The early initiation of ocrelizumab demonstrated optimal efficacy in treatment‐naïve [57] and highly active PwMS [58]. The present study demonstrated the favorable real‐world effectiveness of ocrelizumab, aligning with findings from prior cohorts [30, 59] and clinical trials [55, 60].

### 4.1 Clinical Outcomes of Ocrelizumab

The clinical efficacy findings of this study consist with those reported in clinical trials [55, 61, 62] and the limited real‐world studies conducted to date [28, 55, 63, 64]. Additionally, recent systematic reviews and meta‐analyses have further confirmed the efficacy of ocrelizumab in MS management [8, 65]. The ENSEMBLE study in RRMS demonstrated that most of patients treated with ocrelizumab exhibited an absence of disease activity over 4 years [66]. Evidence indicates that ocrelizumab effectively prevents relapse‐associated disability worsening (RAW), as disability progression in PwMS receiving ocrelizumab was predominantly linked to progression independent of relapse activity (PIRA) [67, 68]. The lower ARR observed in prior trials [55, 61, 62] at 12 months compared to our study may be attributed to the inclusion of PMS alongside RRMS, variations in follow‐up durations, and the real‐world context of our study, which differed significantly from controlled trial settings. In our cohort, PwMS who initiated ocrelizumab as first‐line therapy exhibited greater short‐term improvements in relapse rate and functional measures. However, given baseline differences between groups and the exploratory nature of the subgroup analyses, these findings should not be interpreted as definitive evidence of comparative superiority.

Given that most previous studies evaluated clinical benefits in MS patients using basic measures such as EDSS, there is a need for more qualified, sensitive, and meaningful assessments of disability progression [69]. PwMS who received continuous ocrelizumab treatment have demonstrated significantly lower rates of progression in EDSS scores, 9HPT completion time, and T25FW test performance compared to those who initially received placebo [69, 70]. A study by Epstein et al. reported that 75% of older patients with PMS treated with ocrelizumab exhibited stability in T25FW test performance over 2 years [71]. In SPMS patients, a moderate effect on T25FW and slight stabilization in 9‐HPT performance were observed [72].

DMTs such as interferon‐β and glatiramer acetate, as first‐line treatments in MS, are routinely used to decrease the rate of progression from clinically isolated syndrome to MS [73, 74]. However, the escalation methods can often lead to breakthrough disease activity, prompting treatment changes and less favorable outcomes [75, 76, 77]. Current clinical recommendations emphasizing on the use of high‐efficacy DMTs early to control disease activity and minimize the risk of long‐term disability progression [66]. In addition, treatment transitions in MS need careful consideration, because these changes can significantly increase the risk of disease recurrence [59]. Previous studies that evaluated the clinical outcomes of transitioning from other DMTs to ocrelizumab revealed that ocrelizumab can be a strong therapeutic option for previously treated PwMS that can reduce the risk of disease reactivation [28, 59, 78, 79, 80]. The baseline ARR in our study was in the range reported in a recent systematic review and meta‐analysis of real‐world MS studies, with some included studies had lower baseline relapse rates and others reporting higher values [81]. Additionally, in the current study, the shorter disease duration likely contributed to the higher baseline ARR, as PwMS in the earlier stages of MS commonly exhibit greater inflammatory activity and more relapses.

Ocrelizumab has demonstrated rapid clinical and radiological effectiveness, even after discontinuation of other DMTs [78, 82]. It has high efficacy in PwMS who switch from first‐line injectable agents or highly potent therapies such as alemtuzumab, natalizumab, fingolimod, and cladribine to ocrelizumab [30, 78]. Furthermore, the therapeutic effects of ocrelizumab are not influenced by prior DMTs [83]. A 6‐month study on PwMS revealed that transition to ocrelizumab stabled the disability level in PwMS [78]. This differences in efficacy are likely due to the intrinsic superiority of ocrelizumab in controlling disease activity compared with other DMTs, such as fingolimod, rather than variations in the rate of immunosuppression, as both therapies rapidly reduce or redistribute lymphocytes [84, 85]. Given that the effects of anti‐CD20 therapies accumulate over time [86], extended follow‐up periods are warranted to assess differences in confirmed disability progression between PwMS treated with ocrelizumab and other DMTs.

### 4.2 Psychological Outcomes of Ocrelizumab

Clinical trials on ocrelizumab were aimed to evaluate relapse rates and sustained disability progression. However, assessing the secondary outcomes, such as psychological changes improves the comprehensive understanding of the potential therapeutic impact of ocrelizumab [87, 88]. A 12‐month follow‐up study on ocrelizumab showed improvements in fatigue, anxiety, and mood, with the most pronounced effects on mental rather than physical domains [87]. These findings suggest that ocrelizumab may also impact the broader aspects of psychological health and quality of life in PwMS [87]. Importantly, these improvements addressed some of the most challenging aspects of MS, such as fatigue, which is highly prevalent among patients and associated with morbidity [89] and decreased work productivity [90]. However, it has not been sufficiently evaluated in routine clinical practice [87]. In a real‐world 6‐month follow‐up study of RRMS patients, depression, anxiety, and fatigue were decreased following treatment with ocrelizumab [91]. Conversely, a 22‐month longitudinal study reported no significant changes in these psychological parameters after using ocrelizumab in PwMS [92]. These findings highlight the need for a multidimensional approach to MS management that integrates pharmacological therapy with the purpose of improving psychological and social support to optimize patient well‐being and overall outcomes [21].

### 4.3 Safety of Ocrelizumab

Early treatment with high‐efficacy DMTs requires careful consideration of safety, as some high‐efficacy agents are associated with serious AEs, including progressive multifocal leukoencephalopathy and secondary autoimmune disorders [93, 94, 95, 96]. Anti‐CD20 B‐cell‐depleting therapies may increase the risk of infection over time [97]. Upper respiratory and urinary tract infections were the most frequent AEs in trials, while observational studies have reported minor infections with less frequency in PwMS [28, 30, 98]. Consistent with prior research [55, 78], in our study, ocrelizumab was well tolerated in PwMS, with most infections was mild and non‐recurrent, and no severe infections was observed that needs hospitalization. The absence of treatment discontinuations further supports the acceptable safety of ocrelizumab in PwMS. In the ENSEMBLE study, IRRs and infections were the most commonly reported adverse events, while serious events and treatment discontinuations were rare, and no serious safety concerns were identified [66]. In a Chilean study, serious infections occurred in 4.6% of PwMS, with two deaths reported during follow‐up that one of them was due to severe COVID‐19 and the other one was related to metastatic cancer [99]. Although most reported infections associated with ocrelizumab were minor, some viral infections were reported, including fulminant hepatitis linked to echovirus 25 and HSV‐2 encephalitis [100, 101]. While some cases of neoplasms were reported in previous trials [60] and warranting continued evaluation within the context of neoplasm epidemiology in PwMS and long‐term ocrelizumab use [55], no cases were identified in our follow‐up. However, this may be attributed to the small sample size of our study. Prior systematic reviews and meta‐analyses have demonstrated that while ocrelizumab is commonly well tolerated in PwMS [102], rigorous patient monitoring and comprehensive education concerning anti‐CD20 monoclonal antibody therapies like ocrelizumab are imperative for the early identification of adverse events and the reduction of associated clinical risks [103].

The small sample size and single‐center design of our study limit the generalizability of its findings. Although the findings of this study on the efficacy and safety of ocrelizumab in MS consist with previous research, larger multicenter studies are needed to validate these results.

### 4.4 Limitations

This study has some limitations that should be considered during the interpretating of its results. It was conducted at a single center and included a small number of PwMS treated with ocrelizumab, which may restrict the generalizability of its findings. Additionally, there was a lack of imaging data, laboratory tests, and cognitive performance assessments in this study. The lack of MRI monitoring and cognitive function evaluation prevented us from assessing subclinical disease activity, radiological changes, and potential cognitive improvement following ocrelizumab treatment. Thus, the interpretation of therapeutic effects in this study was only based on clinical and functional outcomes. This study has an observational design rather than an RCT, which is the gold standard for assessing the efficacy of DMTs. Although observational studies provide valuable insights by comparing patients to themselves, they may overestimate the effect of treatments. Furthermore, the improvements in psychological outcomes following ocrelizumab may be a results of the prior treatments [88]. Moreover, the small sample size constrained us to perform multivariable adjustments analyses for potential confounders. To mitigate this, subgroup analyses based on the type treatment initiation were conducted. However, the comparisons between groups were not adjusted, and the potential influence of residual confounding factors cannot be entirely excluded. Participants lost to follow‐up had higher baseline depression scores compared with completers, which may have influenced long‐term psychological outcomes. Therefore, changes in depressive symptoms at 24 months should be interpreted cautiously. Additionally, incomplete data collection and participant dropout led to missing values for some functional and psychological measures at T2. Relapse was identifies based on clinical examination without MRI evaluation. It may have introduced variability in the estimation of ARR compared with studies employing MRI evaluation. Another limitation was that the EDSS was not assessed in 12‐ or 24‐week, which precluded us to identify the disability improvement or worsening. Therefore, disability changes were interpreted based on EDSS changes rather than confirmed improvement or worsening. Since AEs were evaluated through patient‐reported symptoms without laboratory tests, mild or asymptomatic infections may have been under‐detected. Another limitation of this study is the absence of analyses by MS phenotype. The small number of PwMS in subgroups limited the ability to perform reliable statistical comparisons. Likewise, the ability to perform sex‐based analyses was limited. Future research with larger sample sizes, participants form multicenter, and extended follow‐up periods are needed to assess the long‐term effects of ocrelizumab. Furthermore, integrating objective clinical and imaging measures with self‐reported outcomes could help reduce potential bias and enhance the accuracy of the findings.

## 5 Conclusions

The findings of this study suggested that ocrelizumab may effectively reduce disease activity and improve the psychological well‐being of PwMS. The findings also support considering ocrelizumab as a first‐line treatment, as it improved functional characteristics while maintaining psychological outcomes comparable to those initially treated with other DMTs. However, comparisons between treatment‐naïve participants and those switching from other DMTs were exploratory and unadjusted, and therefore should be interpreted cautiously. Although the results indicate clinical benefits in reducing disability and improving psychophysical function, the absence of MRI data limits insight into effects on underlying disease activity. More studies with a larger sample size that integrate MRI and clinical outcomes are needed to confirm these preliminary observations.

## Author Contributions

**Majid Jafari:** data curation, writing – review and editing. **Mohammad Yazdan Panah:** conceptualization, methodology, investigation, formal analysis, visualization, writing – original draft. **Saeed Vaheb:** data curation, resource. **Iman Adibi:** conceptualization, writing – review and editing. **Fereshteh Ashtari:** conceptualization, writing – review and editing. **Vahid Sahygannejad:** conceptualization, investigation, resource, writing – review and editing. **Aram Zabeti:** investigation, writing – review and editing. **Omid Mirmosayyeb:** conceptualization, methodology, validation, supervision, project administration, writing – review and editing. All authors reviewed the manuscript and agreed.

## Funding

The authors received no specific funding for this work.

## Consent

Informed consent was secured from all participants in this study.

## Conflicts of Interest

The authors declare no conflicts of interest.

## Declaration of Generative AI and AI‐Assisted Technologies in the Writing Process

During the preparation of this work, the authors used Chat‐GPT in order to improve the readability and language of the manuscript. After using this service, the authors reviewed and edited the content as needed and take full responsibility for the content of the published article.

## Human Ethics

The ethics committee of Isfahan University of Medical Sciences approved the research protocol (IR. MUI. MED. REC.1403.206). All procedures conformed to the ethical standards in the 1964 Helsinki Declaration.

## Transparency Statement

The lead author Vahid Shaygannejad affirms that this manuscript is an honest, accurate, and transparent account of the study being reported; that no important aspects of the study have been omitted; and that any discrepancies from the study as planned (and, if relevant, registered) have been explained.

## Supporting information


**Table S1:** Demographic, clinical and psychological characteristics of PwMS who completed and not completed the follow‐up. **Table S2:** Demographic, clinical and psychological characteristics of PwMS who were treated with ocrelizumab and those who were DMT Naïve or treated with other disease‐modifying therapies at beginning of the study (T0).

## Data Availability

All data generated or analyzed during this study are included in this article. Further enquiries can be directed to the corresponding author.
