# Supplementary material for: Long‐Term Clinical and Psychological Efficacy and Safety of Ocrelizumab in People With Multiple Sclerosis: A Real‐World Longitudinal Study
Source: Health Sci Rep. 2026 Mar 22;9(3):e72142. doi: 10.1002/hsr2.72142 (PMC13097365; doi:10.1002/hsr2.72142)
Supplement: Supplementary file 1 — Table S1: Demographic, clinical and psychological characteristics of PwMS who completed and not completed the follow‐up. Table S2: Demographic, clinical and psychological characteristics of PwMS who were treated with ocrelizumab and those who were DMT Naïve or treated with other disease‐modifying therapies at beginning of the study (T0). [file HSR2-9-e72142-s001.docx]

**Table S1.** Demographic, clinical and psychological characteristics of PwMS who completed and not completed the follow-up.

| Characteristics | Complete Follow-up  (n = 21) | Lost to Follow-up  (n = 30) | Effect Size | P-value |
| --- | --- | --- | --- | --- |
| Age; mean (SD) | 37.3 (10.1) | 39.9 (9.1) | Cohen’s d = -0.27 | 0.341 |
| Age at Onset; median (range) | 29 (15 to 48) | 30.5 (16 to 55) | MWU = 287.5 | 0.598 |
| Sex; female (%) | 19 (90.5%) | 25 (83.3%) | Chi^2^ = 0.53 | 0.466 |
| MS Subtype | RRMS: 15 (71.4%)  PMS: 6 (28.6%) | RRMS: 17 (56.7%)  PMS: 13 (43.3%) | Chi^2^ = 1.15 | 0.283 |
| Disease Duration; median (range) | 7 (3 to 14) | 8.5 (2 to 20) | MWU = 265.5 | 0.338 |
| Baseline EDSS; median (range) | 3 (1.5 to 4.5) | 3.2 (1.5 to 6.5) | MWU = 282.5 | 0.528 |
| Baseline 9-HPT; median (range) | 23.7 (19.2 to 53.6) | 23.7 (18.8 to 37.2) | MWU = | 0.992 |
| Baseline T25FW; median (range) | 6.9 (5.4 to 9.2) | 6.5 (4.9 to 14.5) | MWU = | 0.856 |
| Baseline ARR; median (range) | 0.5 (0.3 to 2) | 0.4 (0.2 to 2) | MWU = | 0.281 |
| Baseline BAI; median (range) | 13 (0 to 40) | 18.5 (1 to 56) | MWU = | 0.052 |
| Baseline BDI; median (range) | 9 (1 to 47) | 14 (2 to 62) | MWU = | **0.011** |
| Baseline FSS; mean (SD) | 36 (11.3) | 40 (12.1) | Cohen’s d = -0.34 | 0.240 |

Significant *p*-values are shown in **bold**. ARR: annualized relapse rate, BAI: Beck Anxiety Inventory, BDI: Beck's Depression Inventory, EDSS: Expanded Disability Status Scale, 9-HPT: nine-hole peg test, MS: multiple sclerosis, SD: standard deviation, T25FW: timed 25-foot walk test.

**Table S2.** Demographic, clinical and psychological characteristics of PwMS who were treated with ocrelizumab and those who were DMT Naïve or treated with other disease-modifying therapies at beginning of the study (T0).

|  | Initiate with OCR  (n = 21) | Initiate with other DMTs  (n = 30) | Effect Size | *P*-value |
| --- | --- | --- | --- | --- |
| Age (year); Mean (SD) | 37.76 (10.25) | 39.63 (9.01) | Cohen’s d = -0.196 | 0.494 |
| Female (%) | 19 (90.5%) | 25 (83.3%) | Chi ^2^ = 0.532 | 0.466 |
| Age at Disease Onset (year); Mean (SD) | 33.24 (10.17) | 28.17 (8.79) | Cohen’s d = 0.541 | 0.063 |
| Married; n (%) | 17 (81%) | 24 (80%) | Chi ^2^ = 0.007 | 0.933 |
| Year of Education; Median (range) | 12 (6 to 18) | 15 (6 to 18) | MWU = 270.5  Z = -0.893 | 0.372 |
| RRMS; n (%) | 10 (47.6%) | 22 (73.3%) | Chi ^2^ = 3.494 | 0.062 |
| Disease Duration (years); Median (range) | 3 (2 to 16) | 12 (5 to 20) | MWU = 57  Z = -4.989 | **< 0.001** |
| EDSS; Median (range) | 3.5 (1.5 to 4.5) | 3 (1.5 to 6.5) | MWU = 285  Z = 0.561 | 0.561 |
| Follow-up Duration (months); Median (range) | 25.5 (24 to 28) | 25.5 (24 to 28) | MWU = 293  Z = -0.432 | 0.666 |
| ARR; Median (range) | 0.42 (0.33 to 2) | 0.43 (0.25 to 0.8) | MWU = 127  Z = -3.621 | **< 0.001** |
| 9-HPT; Median (range) | 25.3 (20 to 37.2) | 23.5 (18.8 to 53.6) | MWU = 290.5  Z = -0.469 | 0.639 |
| T25FW; Median (range) | 7.6 (5.3 to 14.5) | 6.4 (4.9 to 12.2) | MWU = 196.5  Z = -2.268 | **0.023** |
| Anxiety; Mean (SD) | 18.19 (13.18) | 16.1 (10.18) | Cohen’s d = 0.182 | 0.526 |
| Depression; Median (range) | 13 (4 to 62) | 12 (1 to 47) | MWU = 274  Z = -0.785 | 0.432 |
| Fatigue; Mean (SD) | 40.38 (12.15) | 37 (11.59) | Cohen’s d = 0.286 | 0.320 |

Significant *p*-values are shown in **bold**. ARR: annualized relapse rate, DMTs: disease modifying therapies, EDSS: Expanded Disability Status Scale, 9-HPT: nine-hole peg test, MS: multiple sclerosis, OCR: ocrelizumab, SD: standard deviation, T25FW: timed 25-foot walk test.
